# Lumbocaval Shunt for Idiopathic Intracranial Hypertension: A Technical Report and Case Series

**DOI:** 10.1227/neuprac.0000000000000113

**Published:** 2024-10-01

**Authors:** Nanthiya Sujijantarat, Andrew B. Koo, Aladine A. Elsamadicy, Joseph P. Antonios, Daniela Renedo, Joseph O. Haynes, Bushra Fathima, Brianna C. Theriault, Miguel M. Chavez, Abdelaziz Amllay, Kamil W. Nowicki, Matthew Kanzler, Jasmine W. Jiang, Apurv H. Shekar, Ryan M. Hebert, Michael L. DiLuna, Charles C. Matouk

**Affiliations:** ‡ Department of Neurosurgery, Yale University School of Medicine, New Haven, Connecticut, USA;; § Department of Radiology & Biomedical Imaging, Yale University School of Medicine, New Haven, Connecticut, USA

**Keywords:** Idiopathic intracranial hypertension, CSF shunt, Lumbocaval shunt, Lumboatrial shunt, Case series

## Abstract

**BACKGROUND AND OBJECTIVES:**

Neurosurgical management of idiopathic intracranial hypertension (IIH) can be challenging given high rates of revision associated with cerebrospinal fluid shunting. In this study, we present a technical report and early outcomes for lumbocaval shunt (LCS) placement in difficult-to-manage cases.

**METHODS:**

A literature search was performed for previous reports of LCS or lumboatrial shunt. Electronic medical records of patients who underwent placement of LCS for the treatment of IIH at a single institution were reviewed. Based on early experience and outcomes, our modified technique for LCS is described.

**RESULTS:**

Six patients (4 females, median age 36 years [IQR 31-43]) underwent placement of LCS between October 2023 and April 2024. LCS was completed in all cases without intraoperative complications. The median operative time was 88.5 minutes [IQR 79.5-158.8]. One patient developed low-pressure headaches that resolved after the addition of a shunt-assist device. Five of 6 patients reported improved headache at the last follow-up visit, with 4 of 5 patients reporting that their high-pressure headaches completely resolved (median time to the last follow-up of one month [IQR 1-2 months]). During the study period, one shunt revision was performed because of migration of the lumbar shunt into a suprafascial pocket. This led to modification of the surgical technique, specifically the inclusion of anchoring dips.

**CONCLUSION:**

LCS may represent an alternative shunting technique in difficult-to-manage patients with IIH. Further assessment of long-term outcomes is needed.

**ABBREVIATIONS:**
IIH: idiopathic intracranial hypertension
IJV: internal jugular vein
IVC: Inferior Vena Cava
LCS: lumbocaval shunt
LP: lumbar puncture
LPS: lumboperitoneal shunt
OP: opening pressure
OR: operating room.

Idiopathic intracranial hypertension (IIH) is a clinical syndrome consisting of headaches, visual disturbances, and elevated intracranial pressure in the context of normal intracranial imaging.^1^ Despite rapidly evolving understanding of its pathophysiology, management of IIH symptoms remains challenging.^2^ When IIH is refractory to medical management, ventricular or lumbar shunting is indicated to prevent vision loss and improve debilitating, high-pressure headaches.^3^ The distal catheter is most frequently positioned in the peritoneal cavity; however, the right atrium and pleural space are other common locations. Unfortunately, these shunting procedures are associated with high failure rates and poor, long-term symptom control.^4-6^ More recently, intracranial venous sinus stenting has emerged as a promising alternative treatment strategy. However, long-term outcomes are uncertain.^7,8^

Lumbocaval shunt (LCS) was first described in 1983 in a patient with multiple shunt failures.^9^ Since this time, there have been several case reports with varying descriptions of LCS.^9-12^ In this study, we present a technical report and early institutional experience with LCS in difficult-to-manage IIH cases.

## METHODS

### Patient Selection

The patient underwent appropriate IIH evaluation before their visit. This included a complete neurological examination, preoperative ophthalmological evaluation, MRI of the brain, and lumbar puncture (LP) with documentation of high opening pressure (OP). Patients were referred to us after the IIH diagnosis was made. LCS was considered in adult patients (≥18 years old) who failed best medical therapy and who had either previous shunt placement or revision, or other factors making traditional shunt placement unfavorable. Femoral vein must be patent. For the purpose of this series, patients with pulmonary hypertension, cardiac failure, or volume overload were excluded.

### Technical Description

Four case reports were found through literature search describing placement of LCS or lumboatrial shunt.^9-12^ Our modified technique for LCS placement is described below.

#### Room Setup and Equipment

A hybrid neurovascular operating room (OR) with a single plane fluoroscopy was used. If this was not available, a standard OR was used with a C-arm fluoroscopy.

In addition to standard OR instruments, we used a 60-cm disposable catheter passer with a 3.3-mm internal diameter (Medtronic; other substitutes may be available). The shunt assembly consisted of a standard lumbar drain kit with a spinal trochar, lumbar drain tubing, and silicone suture dips; a GAV 2.0 Shunt System (Aesculap); and a SHUNTASSISTANT 2.0 Valve (Aesculap). For the femoral venous access, we used a standard 5F micropuncture set, a 145-cm 0.035″ Bentson wire (Cook Medical; other substitutes are available), and a 9F Peel-Away Introducer Set (Cook Medical; other substitutes are available).

#### Prone Stage: Insertion of the Lumbar Catheter

**FIGURE 1.**
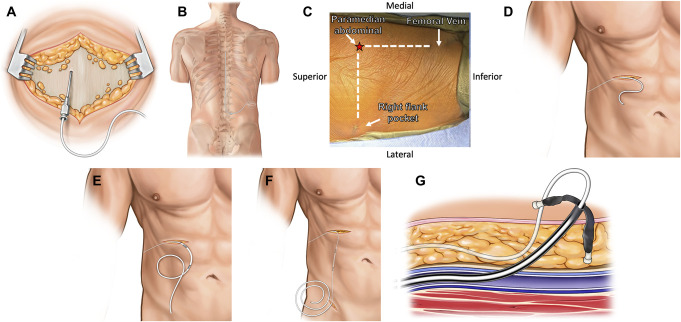
**A** and **B**, Prone stage of the LCS procedure. **C**, The relationship between the flank incision, the abdominal incision, and the femoral vein access site. **D**-**G**, Supine stage of LCS. LCS, lumbocaval shunt.

After induction of general anesthesia, the patient was transferred to the operating table in prone position. After localizing the L2-L3 interspinous space under x-ray guidance, a midline incision was made. A small, suprafascial pocket was created. A spinal trocar was inserted through a paramedian entry point into the desired interspinous space. Using fluoroscopy, a lumbar catheter was inserted into the thecal sac (Figure 1A) with its tip located in the mid thoracic spine.

A disposable passer was used to create a subcutaneous channel extending from the midline incision to the right flank. This passer was reassembled for later use during the supine stage of surgery. A small, suprafascial pocket was created over the right flank. The lumbar catheter was passed through this subcutaneous channel. A silk suture was used to tie off the distal end of the catheter to prevent cerebrospinal fluid (CSF) leakage into the pocket. The catheter was curled up in the right flank pocket (Figure 1B), which was temporarily closed with staples.

The patient was transferred to a stretcher and then back onto the OR table in supine position. The abdomen, right flank, and right inguinal regions were prepped and draped in a standard fashion.

#### Supine Stage: Insertion of the Shunt Catheter into the Inferior Vena Cava

A small incision was made in the right upper quadrant of the abdomen at the level of the right flank pocket. The disposable passer was again used to create a subcutaneous channel extending from the flank incision to the planned abdominal incision. Figure 1C shows the relationship between the flank and abdominal incisions and the site of right common femoral vein access. The lumbar drain catheter was passed through this channel (Figure 1D) and attached to a GAV 2.0 Shunt System (Aesculap). A SHUNTASSISTANT 2.0 Valve (Aesculap) was added to the circuit immediately distal to the shunt valve. Figure 1E shows the final shunt configuration. Next, an ultrasound was again used to confirm the right common femoral vein puncture site in line with the right upper abdominal incision. A small nick incision was made in the overlying skin. A right femoral venous access was obtained using a Seldinger technique similar to a central line insertion. First, the femoral vein puncture was performed using a microneedle, angling the needle in line with the course of the femoral vein. Next, a 5F microsheath, which came coupled with a microintroducer in the micropuncture set, was then used to dilate the femoral vein over the microwire. The microintroducer and the wire were then removed, leaving the microsheath in place. The Bentson wire (Cook Medical) was introduced into the microsheath and navigated into the Inferior Vena Cava (IVC), and the microsheath was carefully withdrawn. Finally, the 9F Peel-Away Sheath (Cook Medical) was introduced over the Bentson wire into the femoral vein.

**FIGURE 2.**
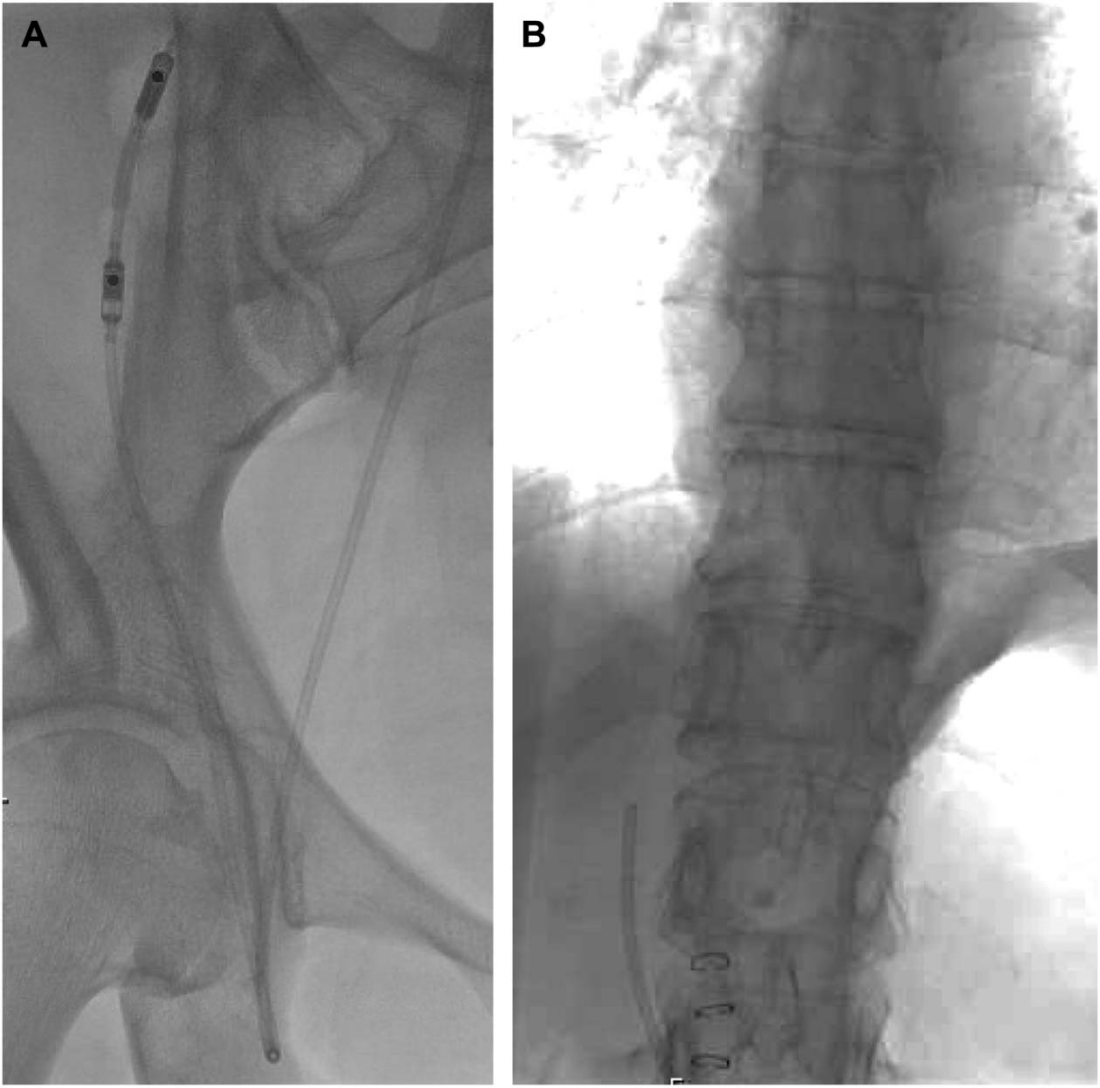
**A**, Fluoroscopic image of the valve and the distal shunt catheter coursing from the upper abdomen down to the femoral vein region before being internalized into the inferior vena cava. **B**, Fluoroscopic confirmation of the shunt tip position proximal to the heart.

**FIGURE 3.**
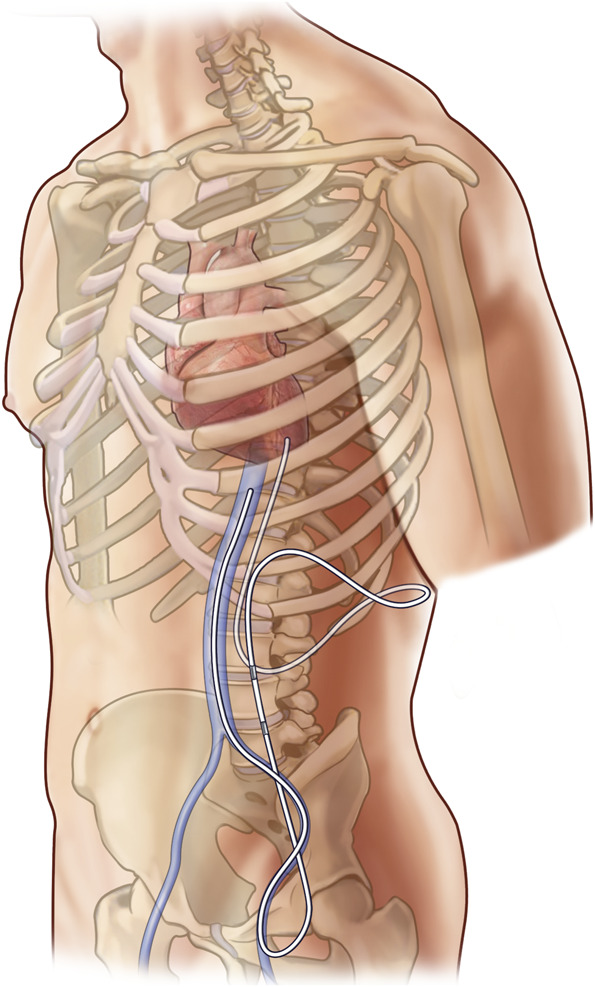
Final lumbocaval shunt configuration.

**FIGURE 4.**
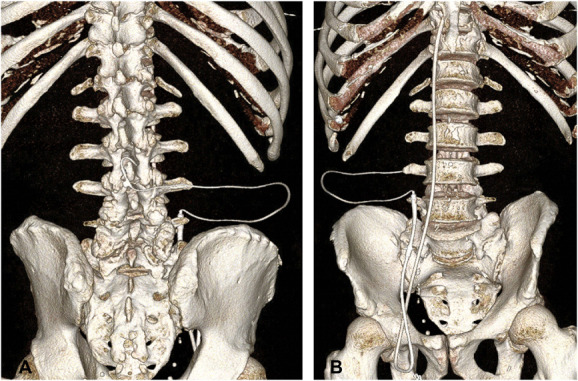
Computed tomography reconstruction with **A**, anteroposterior and **B**, posteroanterior views of the lumbocaval shunt.

After verifying that both valves were inserted in a correct orientation, the shunt assembly was tunneled to the right inguinal region (Figure 1F). We then estimated the length of the intravenous portion of the catheter by measuring the length of the Bentson wire in the IVC with its tip just proximal to the heart (verified by fluoroscopy). The Bentson wire and the introducer of the 9F Peel-Away Sheath were then removed, leaving the sheath in place. The distal catheter was inserted into the Peel-Away sheath and positioned in the IVC, again with its tip just proximal to the heart (Figure 1G). The sheath was then carefully peeled away from the catheter and discarded. Brief manual pressure was held over the groin site to achieve hemostasis before skin closure with a stitch. Figure 2A shows the fluoroscopic image of the valve and the distal shunt catheter coursing from the upper abdomen down to the femoral vein region before being internalized into the IVC. The final shunt tip position was confirmed by fluoroscopy (Figure 2B). Figure 3 shows the schematic of the final shunt configuration. Figure 4 shows a computed tomography reconstruction with anteroposterior (A) and posteroanterior (B) views of the LCS. Video details the steps of the procedure.

#### Postoperative Care

Chest and abdominal x-rays were obtained postoperatively to document the final position of the shunt. Follow-ups were arranged for 2 weeks postoperatively for wound check and again in 1 month for assessment of symptoms. Further follow-up was determined on a case-by-case basis based on the one-month follow-up.

### Institutional Cohort Participants and Clinical Characteristics

This study is a retrospective case series of consecutive patients who underwent placement of LCS for the treatment of IIH at Yale-New Haven Hospital between October 2023 and April 2024. Medical history, demographic information, baseline characteristics, clinical presentation, intraoperative and clinical outcomes were collected from the electronic medical records. An informed consent for the procedure was obtained in all cases per institutional protocol. The participants of any identifiable individuals consented to publication of his/her image. The study is in accordance with the PROCESS guidelines^13^ and was approved by the Institutional Review Board (#2000028154). Descriptive statistical analyses were performed using Microsoft Excel (Version 16.87). The relevant anonymized patient-level data area is available upon reasonable request from the authors.

## RESULTS

### Demographics and Outcomes of the Institutional Cohort

Six patients underwent placement of LCS between October 2023 and April 2024. There were 4 females (67%). The median age was 36 years [IQR 31-43]. The median body mass index was 32 [IQR 24-49]. Symptoms described were headache in all patients and visual obscurations in 4 patients (67%). Two patients (33%) had documented papilledema. All patients had slit ventricles on computed tomography and underwent LP as part of their IIH workup. The median OP was 31 cmH_2_O [IQR 30-41]. Five of 6 patients had a previous shunt procedure, with 4 of 5 having had more than 1 shunt revision.

LCS was successfully completed in all cases without intraoperative complications. The median operative time was 88.5 minutes [IQR 79.5-158.8]. One patient developed low-pressure headaches on standing and required the addition of a gravitational unit (a SHUNTASSISTANT 2.0 Valve [Aesculap]) during the same admission. There was no bleeding complication or dislodgement from the vein. The median length of stay was 4 days [IQR 1-13 days]. The median postoperative day at discharge was 1.5 [IQR 1-6.5]. All patients were discharged home.

At the time of writing, 5 of 6 patients reported improved headaches at the last follow-up visit, with 4 of 5 patients reporting that their high-pressure headaches were completely resolved. The patient who did not have headache improvement at the last follow-up initially reported remarkable relief at the first follow-up visit, but the symptoms returned at the one-month follow-up. The median time to the last follow-up visit was 1 month [IQR 1-2 months]. During this period, 1 patient's lumbar catheter migrated into the suprafascial pocket and required revision. This led to a modification of the surgical technique in subsequent cases, specifically anchoring to the lumbar drain to the fascia using silicone dips.

Of the 2 patients with documented papilledema preoperatively, 1 did not have a neuro-ophthalmological follow-up and 1 had documented improvement in papilledema. Table details patient characteristics and outcomes.

**TABLE.** Patient Characteristics and Outcomes

Cohort characteristics (n = 6)	
Median age, y [IQR]	36 [31-43]
Sex, female, n (%)	4 (67)
Body mass index	32 [IQR 24-49]
Symptoms, n (%)	
Headache	6 (100)
Documented papilledema	2 (33)
Slit ventricles, n (%)	6 (100)
Median opening pressure on lumbar puncture, cmH2O [IQR]	31 [30-41]

Outcomes	
Successful implantation, n (%)	6 (100)
Intraoperative complications, n (%)	0 (0)
Median operative time, min [IQR]	88.5 [IQR 79.5-158.8]
Median length of stay, d [IQR]	4 [1-13]
Median postoperative day at discharge, d [IQR]	1.5 [1-6.5]
Disposition—home, n (%)	6 (100)
Postoperative complications, n (%)	
Infection	0 (0)
Bleeding complications	0 (0)
Thromboembolism	0 (0)
Low-pressure headaches requiring reoperation	1 (17)^a^
Catheter migration	1 (17)^b^
Median time to the last follow-up visit, mo [IQR]	1 [1-2]
Symptomatic relief at the last follow-up, n (%)	
Headache	5/6 (83)^c^
Documented papilledema	1/1 (100)^d^

a One revision of an early patient in our series was due to low-pressure headaches during the hospital stay, requiring addition of a gravitational unit. Since this patient, all shunt systems were modified to include a gravitational unit up front, with no other revision for low-pressure headaches in the series.

b One patient, also early in our experience, was found to have catheter migration into the lumbar suprafascial pocket during the follow-up period. Since this patient, the technique was modified to include anchoring the lumbar catheter with silicone dips in all subsequent patients, with no further revision for catheter migration.

c One patient who did not have headache improvement at the last follow-up initially reported remarkable relief at the first follow-up visit, but the symptoms returned at the one-month follow-up.

d Only one of 2 patients with documented papilledema had neuro-ophthalmological follow-up available.

## DISCUSSION

In this study, we report successful placement of LCS and early outcomes for difficult-to-manage IIH cases. We describe technical aspects of our modified approach.

Classically, the criteria for IIH diagnosis are OP ≥ 25 cmH2O without mass lesion.^1^ Patients with IIH typically experience headaches and/or vision loss that can be progressive and debilitating in daily life. When medical management fails, a CSF shunt is typically offered to prevent vision loss and alleviate symptoms. However, shunt failure rates in IIH are remarkably high.^4-6^ These high complication rates have led to the increasing popularity of venous sinus stenting. However, the long-term efficacy of this approach remains uncertain. In one recent study, 57% of postvenous sinus stenting patients with IIH experienced symptom recurrence in one year.^8^

Several shunting procedures have been described, the most popular of which include ventriculoperitoneal, ventriculoatrial, ventriculopleural, and lumboperitoneal shunt (LPS). Evidence is conflicting in the superiority of any specific shunting technique in patients with IIH.^2,5,14^ Notwithstanding, LPS has the advantage of avoiding the intracranial space.^15^ Andreão et al suggested that LPS may be better at improving headaches in IIH, but with similarly high failure rates compared with ventriculoperitoneal.^16^

Existing literature is sparse on LCS and is limited to case reports where a conversion of LPS to LCS was performed.^9-12^ In 1983, Friedman and Gass^9^ described a two-stage procedure, consisting of first replacing the proximal catheter at the old laminectomy site in a prone position and tunneling it to the flank which was then closed. In a supine stage, the catheter was tunneled to the upper chest wall and the neck and internalized into the internal jugular vein (IJV). By using a pumpable reservoir, the authors stated that positioning the device over the rib assisted in the evaluation of shunt patency should there be a future need. In 1988, Aoki^10^ described a conversion through a femoral access. The author performed the procedure in a supine position only because the existing lumbar catheter and reservoir in the right abdomen did not have to be revised. In 2006, Pülhorn and Redfern^11^ described a procedure in a supine position by re-exploring the abdominal incision. The catheter was tunneled to the right retroauricular region where it was connected to a valve, and the distal shunt was then inserted into the IJV. The existing proximal lumbar catheter was left intact. Most recently, in 2015, Sankey et al^12^ described a procedure in a lateral decubitus position. The authors first inserted a lumbar catheter into the midline incision, tunneled the catheter to the flank, lateral chest, and neck, and then inserted the distal end into the IJV using ultrasound guidance. To our knowledge, reports of de novo LCS placement do not exist in the literature.

LCS has theoretical advantages over conventional shunts. Similar to LPS, the intracranial space is avoided. In our series, all patients had slit ventricles, making intracranial shunt placement more technically challenging. Moreover, ventricular collapse around the shunt catheter may be an important contributor to shunt failure in this difficult-to-manage population.^17^ Placement of the distal shunt catheter outside of the peritoneal space may also be advantageous. Physiologically, intraabdominal pressure is higher in obese patients.^18^ Because obesity is a common comorbidity associated with IIH, a diminished gradient between the intracranial pressure and intra-abdominal pressure may reduce the effectiveness of CSF drainage.^19,20^ This insight remains speculative because much is lacking in our understanding of the pathophysiology of IIH. Longer follow-up in a larger sample set is needed to determine where LCS fits into the treatment paradigm of IIH.

Several important technical aspects were noted from our early experience. First, the lumbar portion of the catheter is inserted at L2-3. This is done to reserve the lower lumbar levels for LP should the patient present with shunt failure symptoms. Similar to LPS, catheter migration represents a potential complication. Indeed, an early case in our series required shunt revision because of migration of the lumbar shunt into the lumbar suprafascial pocket. This led to a modification of the surgical technique in which the exiting lumbar catheter is anchored to the fascia using silicone dips. While the entirety of the procedure can be performed in a lateral decubitus position, we have chosen to perform LCS in 2 stages to minimize the risks of contamination during tunneling of the catheter and for the ease of femoral vein access and fluoroscopic confirmation of the distal catheter tip. Given that overdrainage is a common complication of LPS,^14^ the addition of a gravitational device is performed in all our IIH LCS cases to help mitigate this risk. Finally, the lumbar shunt valve and gravitational unit work best when positioned vertically. Thus, it is often helpful to note the position of the femoral vein puncture site before tunneling the catheter from the flank to the upper abdomen.

The major drawbacks of LCS include the inability to interrogate the shunt in cases of suspected failure. Suspected over- or underdrainage will lead to an open shunt exploration and revision. Loss of femoral venous access is a consideration that may be more relevant in some patients. While we have not had thromboembolic complications in our series, this is a plausible risk given placement of foreign object into the venous system, similar to ventriculoatrial shunt placement.^21^ Patients with a history of lower extremity deep venous thrombosis or those with especially high risks for venous thrombosis may not be a good candidate for the procedure. Finally, while we have not experienced shunt infection in our series, shunt infection after this procedure can lead to systemic infection and vice versa, because of placement of the distal catheter into the systemic circulation.

### Limitations

The major limitations of our study include a small sample size, retrospective design, and relatively short follow-up time (median of 1 month). Longer term follow-up with sequential fundoscopic examinations may prove to be useful in evaluating effectiveness of LCS. Because of relative novelty, a small number of patients, and the lack of long-term outcomes, it is difficult to determine noninferiority of LCS compared with other traditional shunting procedures. Despite these limitations, LCS may represent an alternative technique in patients who have failed conventional shunting or have higher-than-average risk with standard shunting procedures (such as hostile pleura or abdomen, or factors that increase the risk of intracranial shunt placement such as the presence of existing intracranial hardware, previous infections, or the need for early anticoagulation). Future studies would benefit from examining long-term complications and outcomes in this patient population.

## CONCLUSION

LCS can be performed safely and may represent an alternative technique in difficult-to-manage IIH cases. Long-term outcome is needed to better understand its role in the management of patients with IIH.
